# Not one step backwards in recognition of LGBT+ rights

**DOI:** 10.1002/jia2.26087

**Published:** 2023-05-16

**Authors:** Ricardo Baruch‐Dominguez, Juan Carlos Mendoza‐Pérez, Siobhan Guerrero‐Mc Manus

**Affiliations:** ^1^ Faculty of Medicine UNAM Mexico City Mexico; ^2^ Center for Interdisciplinary Research on Science and Humanities UNAM Mexico City Mexico

1

The International Day Against Homophobia, Transphobia, and Biphobia (IDAHOT) is an occasion that reminds us how violence and discrimination experienced by lesbian, gay, bisexual, transgender, queer, nonbinary and other identities (LGBT+) have adverse effects on health. Homophobia and transphobia are manifested in inequalities ranging from structural to those experienced by individuals [1].

The authors of this article live in Mexico, where the Constitution legally recognizes the right not to be discriminated against, where same‐sex couples can marry and adopt children, and where people living with HIV have the right to access antiretroviral treatment. However, Mexico is also the country with the second highest number of homophobic and transphobic hate murders in the world [2], where being gay or transgender and living with HIV continue to be the leading causes of discrimination in society [3], and where the lack of specific actions for the prevention of HIV in gay men and transgender women results in the highest incidence of new HIV infections being concentrated among these particular groups.

North of our border in the United States, where the “gay liberation” movement began, several states are seeking to restrict sex education in schools, prohibit transgender children from freely developing their personalities, and even ban drag shows, whose primary purpose is to entertain [4]. Anywhere these measures are implemented, LGBT+ people's health will be negatively impacted, and the HIV epidemic will likely continue to grow.

Even in countries with progressive laws, homophobia and transphobia are still alive. In Latin America, there are already eight countries where marriage equality is a reality and many others where the identity of transgender people is legally recognized. However, within the region, violence and discrimination against LGBT+ people are still present in all spheres of life, often sparked by religious movements, conservative politics, and, more recently, by transgender‐exclusionary groups within feminism [5].

The wave of attacks against the rights of LGBT+ people has been felt on all continents. In Burundi, a group of men who have sex with men was arrested during a seminar organized by an HIV prevention organization; in Indonesia, the public activity of LGBT+ people has been strictly limited by local authorities; in Russia, laws discourage public speaking about sexual diversity, which is considered “propaganda”; in China, the government has started cracking down on safe spaces and apps that used to be popular among LGBT+ people [6].

The hostile situation in dozens of countries towards LGBT+ people exposes them to social vulnerability, leading to health problems. Specifically, in virtually all parts of the world, including places with generalized epidemics, HIV prevalence is highest among men who have sex with men and transgender women [7]. There are numerous economic injustices related to homophobia and transphobia and their effects on job accessibility. This shows that the vulnerability of people with diverse sexual orientations and gender identities is closely related to sexual health problems.

Structural homophobia and transphobia are responsible for the lack of positive representation in media, the lack of diverse content in sexuality education provided in schools [8], the lack of sensitization of health personnel to provide friendly services to LGBT+ people [9] and the criminalization of same‐sex sexual practices and gender expressions that disrupt traditional sex roles [10]. At the individual level, homophobia and transphobia are expressed within the family, school and work environments. The resulting mental health effects can be associated with increased suicidal ideation, stress and anxiety, as well as internalized homophobia and transphobia, leading to increased vulnerability to HIV, sexually transmitted infections and substance abuse [11].

The current increase in sexualized drug use—or chemsex—among gay and bisexual men worldwide also contributes to increases in HIV and other infections, such as hepatitis C. Problematic drug use may be associated with experiences of discrimination and violence related to the rejection of their sexual orientation [12]. In the case of transgender people, particularly transgender women, the educational, economic and social precariousness derived from transphobia places them at exceptionally high risk of contracting HIV [13] and experiencing multiple other difficulties, such as substance abuse and lack of access to health services, including those related to mental health and gender‐affirming treatments [14].

The recent Mpox outbreak reminded us that stigma and discrimination exist even in the most LGBT+‐inclusive countries, and that affected communities can make a positive difference in collectively responding to health issues that impact them. Gay and bisexual men rapidly mobilized to face this new challenge, just as was done 40 years ago to respond to HIV in the face of slow government action and indifference [15].

The problem is that communities cannot mobilize if criminalized and persecuted. Nor can they do so if they do not have the resources to have a place to live or to feed themselves. This is why breaking down structural and individual barriers that prevent LGBT+ people from organizing and defending their rights is necessary. Every step backwards in human rights will be a step back in the response against HIV. The scientific community must join with LGBT+ communities and human rights defenders worldwide to prevent gains from being lost and to prevent homophobia and transphobia from being further entrenched in our societies.

Not one step backwards! Or as we say in Spanish, *“iNi un paso atrás!”*

## COMPETING INTERESTS

The authors declare no competing interests.

## AUTHORS’ CONTRIBUTIONS

All authors contributed with ideas, drafting and review of the article.
